# The Impact of Hemodialysis Frequency and Duration on Blood Pressure Management and Quality of Life in End-Stage Renal Disease Patients

**DOI:** 10.3390/healthcare5030052

**Published:** 2017-09-02

**Authors:** Mohammad Ali Shafiee, Pouyan Chamanian, Pouyan Shaker, Yasmin Shahideh, Behrooz Broumand

**Affiliations:** 1 Division of General Internal Medicine, Department of Medicine, Toronto General Hospital, Toronto, ON M2G 2C4, Canada; chamanip@mcmaster.ca (P.C.); pshaker@uwo.ca (P.S.); yasmin.shahideh@gmail.com (Y.S.); 2 Pars Advanced and Minimally Invasive Manners Research Center, Pars Hospital Department of Nephrology, Iran University of Medical Sciences, Tehran 1449614535, Iran; v4broumand@yahoo.com

**Keywords:** hemodialysis, blood pressure, End Stage Renal Disease, quality of life

## Abstract

Cardiovascular complications are the most prominent causes of morbidity and mortality among chronic kidney disease (CKD) and end-stage renal disease (ESRD) patients undergoing standard hemodialysis (HD) therapy. Cardiovascular disease risk is increased significantly through persistent hypertension and blood pressure (BP) fluctuation, which are the most common complications of CKD. It was hypothesized that an extended approach with lengthier and more frequent dialysis sessions, referred to in this paper as “extended hemodialysis” (EHD), can potentially lower and stabilize blood pressure, and consequently reduce the rate of morbidity and mortality. A greater reduction of volume (salt and water) with higher frequency can improve patient quality of life (QOL). Eleven papers, including clinical trials and systematic reviews were chosen and analyzed. The extracted data was used to evaluate the change in blood pressure levels between standard HD and EHD. Overall, the studies showed that EHD resulted in improved blood pressure management; therefore, we concluded that there will be a decrease in cardiovascular disease risk, stroke, and morbidity and mortality rate. There will be also an improvement in patient QOL due to beneficial effects of the EHD.

## 1. Introduction

Chronic kidney disease (CKD) and end-stage renal disease (ESRD) are two main global health concerns with prevalence as high as 11–13% and 0.1% in the general population, respectively [1]. The costs of treatment procedures are extremely high, annually amounting up to $42 billion dollars for hemodialysis (HD) in the United States alone, which accounts up to 9% of all Medicare payments [2,3]. According to the 2015 US Renal Data System Annual Data Report, in 2014, 87.9% of all incident cases began renal replacement therapy with hemodialysis, 9.3% started with peritoneal dialysis, and 2.6% received a pre-emptive kidney transplant [4]. This paper will only focus on HD, due to the high prevalence of this treatment. Standard HD consists of three sessions a week with a duration of about four hours per session [5]. Patients receiving this form of treatment have high hospitalization rates with a long duration of stay. The survival rates have not improved in the past two decades, mainly owing to the inadequacy of this treatment [6]. It is clear that, with such high costs, patients should be receiving a higher quality of HD.

Likewise, the patient quality of life (QOL) does not follow up to the expectations set by the massive burden of resources required for managing CKD and ESRD. Previous studies have reported a poorer QOL in patients with ESRD than those with other chronic diseases, and even cancer [7]. In addition, low QOL scores are affiliated with higher rates of mortality and morbidity [8,9]. US Renal Data System Annual Data Report (2015) indicate mortality rates in ESRD patients undergoing standard HD to be 16.9% [4]. This number can be even higher in less developed countries such as India, where the annual mortality rate comes close to 20% in ESRD patients [10]. Significantly poor QOL suggests that the current standard HD treatment applied worldwide is inadequate.

Hypertension is a common complication of CKD and persists among most patients (80–90%) with ESRD on maintenance HD [11,12,13]. It is also a major risk factor for cardiovascular morbidity and mortality, totaling at around 45% of the deaths in HD patients from 2003–2005 [14]. Increased peripheral vascular resistance and effective circulatory volume are the key determinants of high BP in HD patients, the latter having a more important role due to the limited ability of ESRD patients in excreting extraneous fluid [11,15]. In contrast to standard HD, “extended hemodialysis” (EHD) is defined as having a longer duration and/or more frequent sessions per week. The deleterious impact on the myocardium can thereby be reduced due to shorter inter-dialytic periods and diminished fluid shifts. By normalizing extracellular fluid volume, BP can become more normotensive, thus decreasing the severity of hypertension consequences in chronic patients [16]. We can expect that EHD will more closely mimic the continuous filtration of a normal kidney compared to the current standard HD, leading to reduced BP fluctuations. We hypothesized that removing extra volume through more efficient and frequent HD can improve BP control and therefore reduce cardiovascular risk and improve patients’ QOL.

## 2. Materials and Methods

A search of available literature was done in Medline and Embase to Week 4 of June 2017. The Cinahl and Cochrane databases were searched for systematic reviews. The search terms selected were “hemodialysis” or “dialysis,” and “daily” or “extended” or “frequent” or “home,” combined with “hypertension” as an MESH term using the Boolean search operator AND. Animal and non-English studies were excluded. We found 137 related papers. We reviewed abstracts manually for relevancy to the study topic. Papers were included if they dealt with short daily HD, daily HD, or nocturnal HD, and their effects on hypertension as an outcome of interest. The included study designs were clinical trial and systematic reviews. We excluded all case reports including studies with fewer than five subjects, editorials, or studies that did not include a comparator group. We reduced our scope to eleven papers. After critically analyzing the results of the articles found, we used a logical step-by-step approach in making inferences regarding the management of hypertension and quality of life in ESRD patients.

## 3. Results

Table 1 shows seven clinical trials that compare the effect of standard HD and EHD on hypertension. In all 11 studies identified, blood pressure control parameters were the primary or secondary outcome measures.

Kotanko et al. measured the effects of frequent HD on BP in the randomized controlled Frequent Hemodialysis Network Trials. These trials are the largest published randomized trials of frequent HD, permitting robust evaluations of these interventions on BP. The results of these trials showed that frequent HD reduced BP in both frequent in-center HD and with frequent nocturnal HD sessions at home. The daily trial lowered pre-dialysis systolic BP by −7.7 mmHg [95% CI: −11.9 to −3.5] and diastolic BP by −3.9 mmHg. In the Nocturnal Trial, compared to 3× weekly HD, two months of frequent HD lowered systolic BP by −7.3 mmHg and diastolic BP by −4.2. In the randomized controlled trial by Culleton et al., 2007, pre-dialysis systolic BP in a 6-month follow up decreased in patents randomized to nocturnal HD by 7 mmHg and increased in patients randomized to current standard HD by 4 mmHg with a mean difference of 11 mm Hg (95% CI: −2 to 24 mm Hg). Zimmerman et al., 2014 also found a statistically significant decrease in systolic BP from study entry to the end of the run-in phase (3 months; 151 vs. 138 mmHg; *p* = 0.004).

Table 2 shows systematic reviews and/or meta-analyses of the comparison of conventional hemodialysis and nocturnal hemodialysis. A meta-analysis of 35 study arms of 928 analyzable patients by Susantitaphong et al. indicated a significant decrease in systolic BP in frequent or extended hemodialysis (−14.1; 95% CI, −17.2 to −11.0 mm Hg; *p <* 0.001), and an alteration of diastolic BP by −7.1 mm Hg (95% CI, −9.2 to −4.9; *p* < 0.001). In a systematic review and meta-analysis by Suri et al. (2006), 10 of 11 studies suggested improvements in blood pressure in DHD.

## 4. Discussion

In this review, we found a significant trend in BP improvement among patients undergoing EHD in comparison to those undergoing the current standard HD. The included studies varied considerably with respect to patient population, dialysis time, frequency, location, methods of outcome assessment, and follow-up times. The lowering of inter-dialytic BP for daily HD is consistent. In order to illustrate the importance of a normotensive BP, it is important to outline the basic physiology of BP control and how it is affected by standard HD and EHD. EHD results in a decrease in inter-dialytic period, lower intradialytic BP fluctuations, and increased duration of volume removal per session, all leading to a variety of benefits.

### 4.1. Decreased Intra-Dialytic Solute and Volume Shift

In the case of short HD duration as seen in standard HD, the fast removal of solutes from the patient’s intravascular space results in an abrupt decrease of extracellular osmolality. This causes fluid shift into the interstitial and intracellular space, which may lead to brain edema. Increasing the HD frequency will reduce solute/toxin accumulation. This, in conjunction with a longer period of HD, will reduce the intra-dialytic solute shift and provide more time for equilibration. A less significant shift also means that the patient will have fewer symptoms, less nausea, and an increased appetite. This allows for a more balanced diet, and will not require the patient to drink as much water in order to replenish the decrease in plasma volume.

### 4.2. Reduced Inter-Dialytic Volume Removal

Patients undergoing standard HD can have an estimated intake of 1–2 L of water per day [17]. A standard HD frequency of 3 times a week will then result in a patient’s inter-dialytic weight gain of around 3–6 kg. This excess volume must then be removed in a matter of only 4 h. A lower duration between dialysis sessions in EHD means that there will be a decrease in the amount of inter-dialytic fluid accumulation. This means the removal of fluid during HD creates a less drastic fluid shift between the intracellular, interstitial, and extracellular space. Excessive salt and water play a critical role in raising the BP of HD patients [18,19]. Since cardiovascular complications are the major causes of mortality and morbidity in HD patients, salt and water can be considered as the most important “uremic toxins,” because their retention is of huge concern and has a great impact on the lives of patients. In EHD, a lower fluctuation of volume gain and volume loss causes less renin–angiotensin–aldosterone system (RAAS) activation and catecholamine release [20]. This decreases the shear stress on vascular walls and promotes more compliant vasculature, resulting in fewer atherosclerotic changes and reduced risk of vascular disease.

### 4.3. Myocardial Effect

Finally, through the aforementioned mechanisms, the myocardium will be subjected to reduced toxic effects and stress, additionally decreasing the risk of cardiovascular disease. Inter-dialytic potassium accumulation between two sessions of standard HD followed by rapid potassium removal in a 4 h session could lower the resting membrane potential of cells, leading to significant changes in myocyte excitability, and an increased risk of cardiac arrhythmia [21]. However, gradual potassium removal with shorter inter-dialytic time will lower this risk. The increase in HD duration is also beneficial specifically in reducing the risk of coronary calcification. HD patients commonly have hypocalcemia and hyperphosphatemia. In standard HD, the rate of calcium concentration correction will be faster than that of phosphate concentration correction, owing to phosphate’s low diffusibility. Rapid rise in calcium concentration while phosphate concentration is high can increase the calcium phosphate precipitation risk and lead to a higher chance of coronary calcification in standard HD [22,23,24]. The longer dialysis duration of EHD can help correct this complication.

### 4.4. Quality of Life

The physiological improvements that can be seen and inferred in EHD patients can also play a large role in determining the QOL of CDK and ESRD patients. There are several factors we can examine that will impact the patient QOL in an objective manner, and are improved through the use of EHD. One of the most important factors is the patient’s inter-dialytic weight gain, where a higher inter-dialytic weight gain is shown to significantly increase diastolic BP in patients [24]. More serious symptoms include pulmonary congestion with signs of shortness of breath, orthopnea, pulmonary edema, gastrointestinal congestion with signs of dyspepsia, and loss of appetite. Additionally, weight gain can contribute to the swelling of the legs, due to an increase in interstitial fluid buildup in the feet. This is essentially caused by of the addition of 3 L of volume to each foot, due to the sinking of fluid to the lower extremities [25]. As previously mentioned, the sudden and intense decrease in plasma volume during dialysis can put patients in severe post-dialytic stress, mainly inducing symptomatic hypotensive episodes with symptoms of nausea and vomiting [26]. Brain cell swelling can also occur, leading to symptoms of headache, confusion, seizure, and dizziness. The shorter inter-dialytic period and longer dialysis duration observed during EHD can be expected to alleviate these complications to a certain extent, thus making the patient more functional, which contributes to an objectively improved QOL. As discussed before, it is important to note that, in current standard HD, a large inter-dialytic weight filtered in such a short duration will lead to intense post-dialysis drops in blood volume, which can leave the patient exhausted, jaded, and bed-ridden. EHD can be very beneficial in this sense, since the fluctuations in blood volume will not be as severe, and the patient will be more productive. Patients who are more normotensive as a result of EHD can potentially have a more flexible diet, specifically through less restricted fluid intake due to lower levels of uremic toxins. Patients able to have greater nutrition through higher frequency HD will have an improved QOL, specifically due to the influence of malnutrition on morbidity and mortality rates [27]. Overall, these combined factors will contribute to a greatly enhanced QOL for CKD and ESRD patients.

### 4.5. Cost-Effectiveness

One may argue that the outlined improvements will not compensate for the increased cost and patient access issues. However, the current data indicates that the annual costs required for daily and nocturnal HD are significantly reduced in comparison to standard HD [28]. This is mainly due to reduced nursing, reduced hospitalization rates, and fewer health care expenses. However, even through an increase of in-center HD hours, it should be evident that the initial costs of increased dialysis treatment will make way for the benefits of having a more functioning population of ESRD patients compared to the current population of non-functioning patients. In addition, reductions in drug administration will reduce the cost of treating patients [29]. In terms of issues with access and patient compliance, it can be said that increasing the frequency and duration of HD, as well as executing good technique, will decrease turbulent flow. This will be beneficial as it will reduce access complications. Though the majority of HD patients use catheters, the use of newer techniques for fistula patients can help overcome the drawbacks of more frequent puncturing. Lastly, patient education can be utilized to promote greater patient compliance to EHD.

## 5. Conclusions

In conclusion, the use of EHD can improve patient QOL, due to decreased myocardial stress through lower inter-dialytic weight gain, lower blood pressure fluctuations, and lower and more stable blood pressure. It is essential to raise awareness amongst the public and doctors about the benefits of longer and more frequent HD methods. In addition, serious reconsiderations must be made regarding the current implementation of EHD due to its beneficial impact on BP control, cardiovascular health, and patient QOL. Considering the significance and prevalence of ESRD, and its impact on society, we can hope that future studies and sustainable EHD models will further establish the importance and application of increasing dialysis hours and frequency.

## Figures and Tables

**Table 1:** Summary of clinical trial studies on the effect of “extended hemodialysis” (EHD) on blood pressure.

	First Author/Year	Setting	Study Design	Study Duration	Population, Intervention & Comparison Group	Result	Conclusion
1	Kotanko et al., 2015	Randomized Multicenter Frequent Hemodialysis Network Trials/USA	Randomized, prospective trials	12 months	The Daily Trial randomized 245 patients to 12 months of 6× (“frequent”) versus 3× (“conventional”) weekly in-center hemodialysis. The Nocturnal Trial randomized 87 patients to 12 months of 6× weekly nocturnal hemodialysis versus 3× weekly predominantly home-based hemodialysis	In the Daily Trial, compared to 3× weekly hemodialysis, two months of frequent hemodialysis lowered pre-dialysis SBP by −7.7 mmHg (95% CI: −11.9 to −3.5) and DBP by −3.9 mmHg (95% CI: −6.5 to −1.3). In the Nocturnal Trial, compared to 3× weekly hemodialysis, two months of frequent hemodialysis lowered SBP by −7.3 mmHg (95% CI: −14.2 to −0.3) and DBP by −4.2 mmHg (95% CI: −8.3 to −0.1).	Compared to 3× weekly HD, 6× weekly HD produced a comparable reduction of BP in both the Daily and Nocturnal Trials, indicating that frequent HD reduces BP in both frequent in-center HD and with frequent nocturnal HD sessions at home.
2	Zimmerman et al., 2014	Ottawa Hospital/Canada	Randomized crossover trial	9 months	19 patients were included in the systolic blood pressure analysis	After 3 months of short daily, compared to 3 months of conventional hemodialysis, systolic and diastolic blood pressures were not statistically different (*p* = 0.39, *p* = 0.56 respectively).	Patients treated with short daily HD compared to conventional HD require fewer anti-hypertensive medications to achieve the same blood pressure.
3	Chan et al., 2012	Multicenter	Randomized, prospective trial	Mean time of follow-up 3.1 ± 1.8 years	Total = 42 patients with ESRD were followed before and after conversion to NHD and compared with 29 normal subjects	SBP tended to fall (from 132 ± 20 to 124 ± 14, *p* = 0.07) and DBP fell (from 81 ± 11 to 75 ± 10, *p* = 0.01) after conversion to NHD.	These finding adds to the emerging benefits of frequent hemodialysis by quantifying LV mechanics and blood pressure before and after conversion to NHD.
4	Rocco et al., 2011	Frequent Hemodialysis Network (FHN) Nocturnal Trial/multicenter	Randomized prospective trial	1 year	Total 87 patients were randomized: 42 in the CHD three times per week arm and 45 in the frequent NHD, six times per week.	Mean change of SBP; −9.7; 95% CI: −16.9 to −2.5	Frequent nocturnal hemodialysis improved control of hyperphosphatemia and hypertension.
5	Culleton et al., 2007	Canada/University of Calgary and the University of Alberta	2-group, parallel, randomized controlled trial	2 years	A total of 52 hemodialysis patients randomly assigned in a 1:1 ratio to receive nocturnal hemodialysis 6 times weekly or conventional hemodialysis 3 times weekly.	6-month SBP decreased in patients randomized to NHD by 7 mmHg and increased in patients randomized to CHD by 4 mmHg (mean difference, 11 mm Hg; 95% CI, −2 to 24 mm Hg).	Compared with CHD, NHD reduced blood pressure and improved measures of mineral metabolism.
6	Chan, 2005	Toronto General and Humber Regional Hospitals/University of Toronto/Canada	Before & after study	2 months	10 ESRD patients who switched from conventional to nocturnal hemodialysis	Blood pressure fell, by 23/16 mm Hg after switching to nocturnal hemodialysis Mean change of SBP = from 143 ± 4 to 120 ± 6 mmHg (*p*-value = 0.001) Mean change of DBP = from 86 ± 5 to 70 ± 5 mmHg (*p*-value = 0.02)	Two months after the frequency, duration, and dose of dialysis were increased in these hypertensive ESRD patients, by conversion from conventional hemodialysis to nocturnal hemodialysis, there was a substantial fall in blood pressure
7	Fagugli et al., 2001	Italy	Randomized two-period crossover	6 months	12 patients switch from conventional to daily hemodialysis	SBP on CHD =148.9 ± 14.7 SBP on DHD =126.2 ± 13.3 DBP on CHD = 76.3 ± 5.8 DBP on DHD = 72.1 ± 6.2	This prospective crossover study confirms that DHD allows optimal control of BP

SMD: standardized mean difference; SBP: systolic blood pressure; DBP: diastolic blood pressure; MAP: mean arterial pressure; NHD: nocturnal hemodialysis; CHD: conventional hemodialysis.

**Table 2:** Summary of systematic review and meta-analysis studies on the effect of EHD on blood pressure.

	First Author/Year	Data Bases and Search Strategy	Study Design	Population, Intervention & Comparison Group	Result	Conclusion
1	Liu et al., 2017	Medline, Embase, and the Cochrance Central Register of Controlled Trials for studies up to January 2016 using “nocturnal”, “dialysis”, “hemodialysis”, and “controlled trials” as the search keywords.	Systematic review & meta-analysis	Total 22,508 patients from 28 studies.	SBP: Random model: SMD (NHD Versus CHD): −0.33; 95% CI: −0.49 to −0.18; *p* < 0.001, Fixed model: SMD: −0.17; 95% CI: −0.24 to −0.1; *p* < 0.001 DBP: SMD: −0.32; 95% CI: −0.48 to −0.15; *p* < 0.001, MAP: SMD: −0.69; 95% CI: −1.19 to −0.19; *p* = 0.007	In ESRD patients in cardiovascular associated results such as blood pressure, NHD is superior to CHD.
2	Susantitaphong et al., 2012	Medline literature search (inception–April 2011), Cochrane Central Register of Controlled Trials and ClinicalTrials.gov using the search terms “short daily HD”, “daily HD”, “quotidian HD”, “frequent HD”, “intensive HD”, “nocturnal HD”, and “home HD”.	Systematic review & meta-analysis	Total 928 analyzable patients from 35 study arms assessed SBP	Frequent or quotidian HD resulted in a significant reduction in SBP (−14.1 mmHg; 95% CI: −17.2 to −11.0; *p* < 0.001). DBP changed by −7.1 mm Hg (95% CI, −9.2 to −4.9; *p* < 0.001); MAP changed by −11.8 mmHg (95% CI, −13.8 to −9.8; *p* < 0.001).	Conversion from conventional HD to frequent or quotidian HD is associated with an improvement in cardiac function, and blood pressure parameters.
3	Suri et al., 2006	Medline (OVID 1966 to 31 May 2005) and Embase (OVID 1980 to 31 May 2005), hand-searched reference lists of all potentially relevant articles, reviews, and HD journals	Systematic review	268 subjects from 29 included articles	Ten of 11 studies suggested improvements in blood pressure in DHD. Polling of effect measure not done.	Randomized trials with adequate statistical power are required to establish the efficacy and the safety of DHD.
4	Walsh et al., 2005	Medline (1966 to week 4 of July 2003), Embase (1980 to week 4 of July 2003), Cochrane databases, BioAbstracts, Cinahl, DARE, Health Technology Assessment Database, and Proceedings First using search terms: “nocturnal” or “nightly” as keywords in titles or abstracts, and “dialysis”, “hemodialysis” or “renal dialysis” as MESH or keywords.	Systematic review	Fourteen studies were identified including pre-post within-patient comparison or case control studies. Study sample sizes ranged from 5 to 63 NHD patients.	No pooled data presented	NHD is a potential alternative to conventional thrice weekly hemodialysis.
